# Supplementary material for: Topokaryotyping demonstrates single cell variability and stress dependent variations in nuclear envelope associated domains
Source: Nucleic Acids Res. 2018 Sep 12;46(22):e135. doi: 10.1093/nar/gky818 (PMC6294560; doi:10.1093/nar/gky818)
Supplement: Supplementary Data [file gky818_supplemental_files.pdf]

Supplementary information for

Topokaryotyping demonstrates single cell variability and stress dependent variations in nuclear envelope associated domains

by Anamarija Jurisic, Chloe Robin, Pavel Tarlykov, Lee Siggens, Brigitte Schoell, Anna Jauch, Karl Ekwall, Claus Storgaard Sørensen, Marc Lipinski, Muhammad Shoaib*, Vasily Ogryzko.

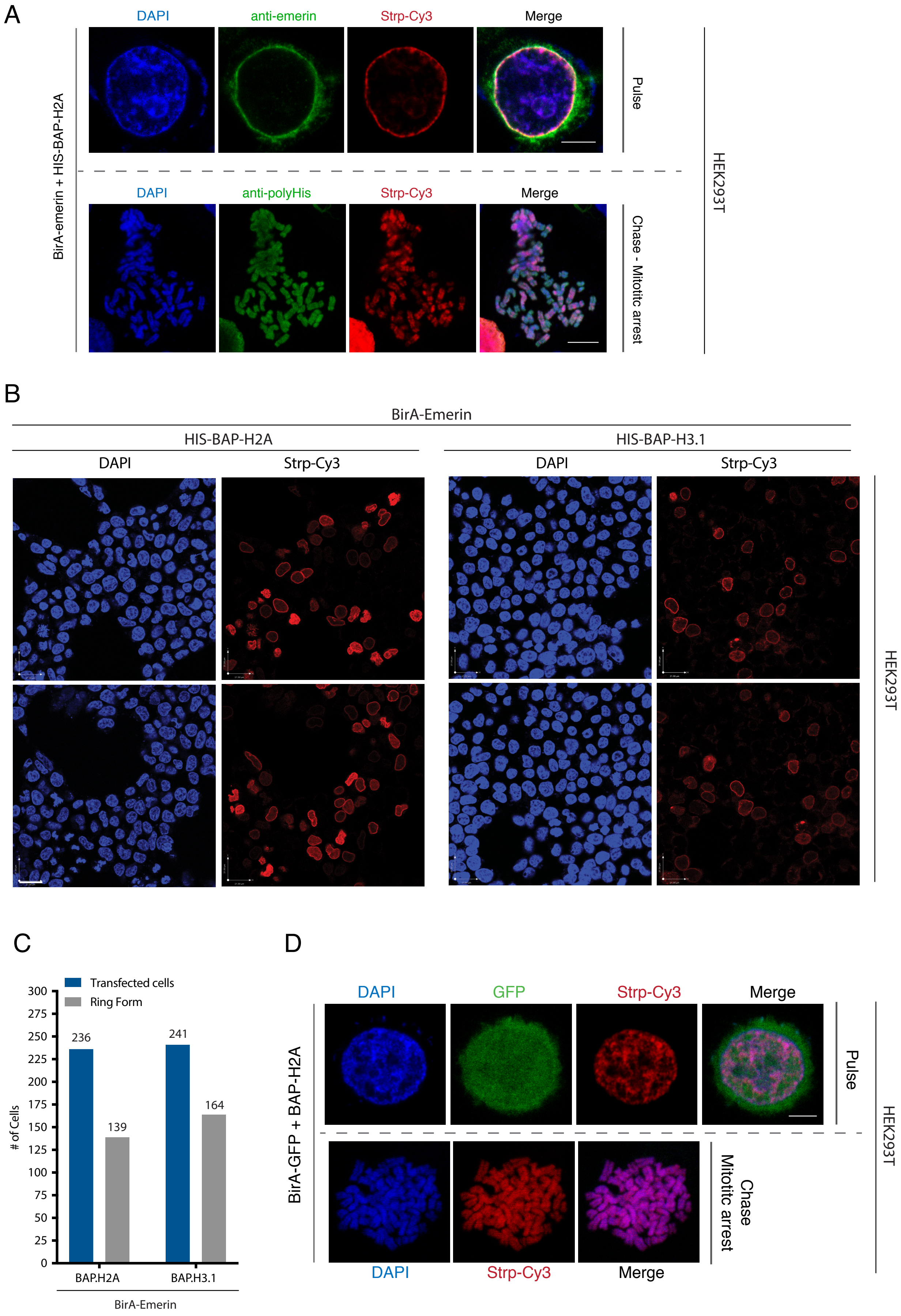
A
DAPI
anti-emerin
Strp-Cy3
Merge
Pulse
BirA-emerin + HIS-BAP-H2A
DAPI
anti-polyHis
Strp-Cy3
Merge
Chase - Mitotitc arrest
HEK293T
B
BirA-Emerin
HIS-BAP-H2A
HIS-BAP-H3.1
DAPI
Strp-Cy3
DAPI
Strp-Cy3
HEK293T
C
Transfected cells
Ring Form
# of Cells
300
275
250
225
200
175
150
125
100
75
50
25
0
236
139
241
164
BAP.H2A
BAP.H3.1
BirA-Emerin
D
DAPI
GFP
Strp-Cy3
Merge
Pulse
BirA-GFP + BAP-H2A
DAPI
Strp-Cy3
Merge
Chase
Mitotitc arrest
HEK293T

S1: **Biotin labeling is NE marker specific.**

(A) Biotinylation takes place at the NE where BirA-Emerin is localized. HEK293T cells were transfected with BirA-emerin and BAP-H2A expressing plasmids, biotin pulse labeled and stained with anti-emerin antibody (green), streptavidin-Cy3 (red) and DAPI (blue; top). Heterogenous distribution of biotin signal and homogenous distribution of BAP-H2A regardless of its biotinylation state along the chromosomes. After the biotin pulse, cells were arrested in mitosis and stained with DAPI to reveal total DNA, streptavidin-Cy3 to reveal biotin and anti-His antibody to detect the His tags present on BAP-H2A histones (regardless of their biotinylation status; bottom). Scale bar: 10 µm. **(B)** Two wide-field images from the experiment in (A) showing the entire viewing field comprising of many transfected cells in both BAP-H2A and BAP-H3.1 expressing cells. Scale bar: 21 µm. **(C)** Quantification of transfected cells with ring-stained pattern **(D)** Homogenous distribution of the biotinylated BAP-H2A when using BirA-GFP. The HEK293T cells were transfected with BirA-GFP and BAP-H2A expressing plasmids and either stained in interphase or arrested in mitosis prior to streptavidin-Cy3 and DAPI staining. GFP signal (green) indicates that BirA-GFP is evenly distributed within the cell and streptavidin-cy3 (red) signal demonstrates the homogenous distribution of biotin signal in the interphase (top) and on mitotic chromosomes (bottom) due to BAP-H2A proximity to BirA-GFP. Scale bar: 10 µm.

A

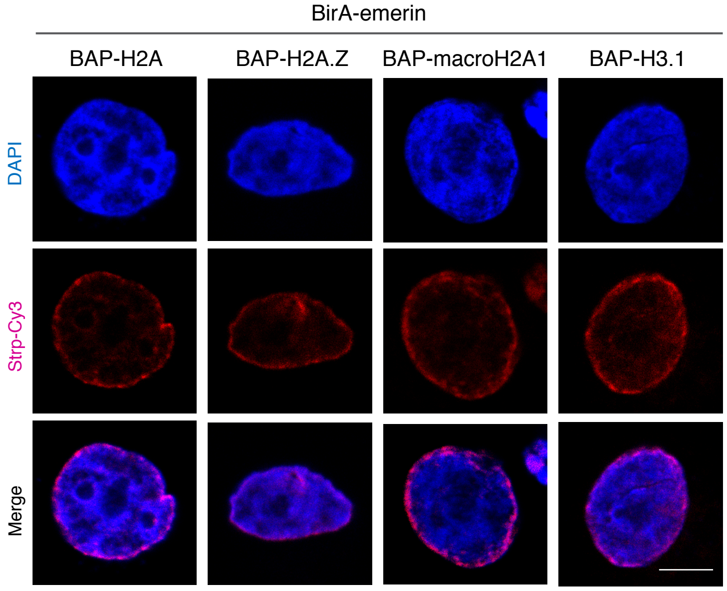

B

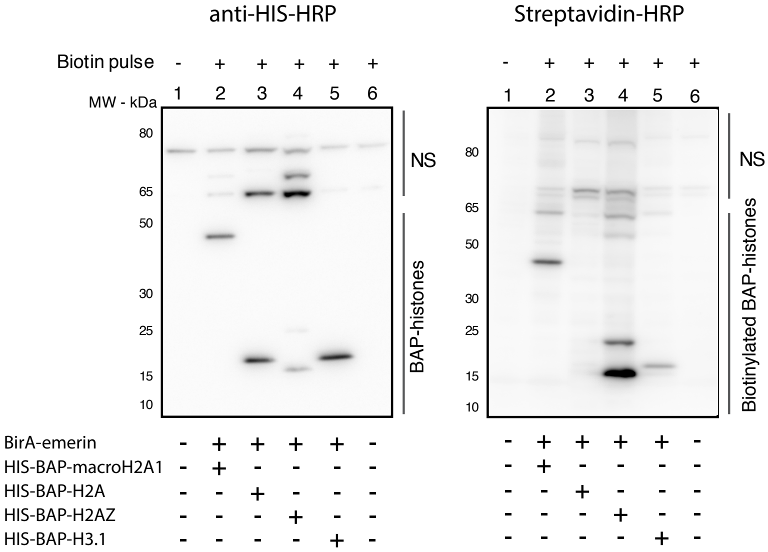

S2: Validation of HeLa cells stably expressing BirA-emerin and BAP-Histones.

(**A**) Biotinylation of NE proximal chromatin in interphase. Hela S3 cell lines stably co-expressing BirA-emerin and BAP-H2A, BAP-H2A.Z, BAP-macroH2A1 or BAP-H3.1 histones. After incubation with biotin for 1h, biotinylated histones were revealed with streptavidin-Cy3 (red) together with counterstaining of DNA with DAPI. Scale bar: 10 µm. (**B**) BAP-histone expression and biotinylation levels. Western blot analysis of nuclei from untransduced and transduced Hela S3 cells using anti-His-HRP (left) or streptavidin-HRP (right) conjugates. The bands corresponding to BAP-histones, biotinylated BAP-histones, and non-specific signals (NS) are indicated.

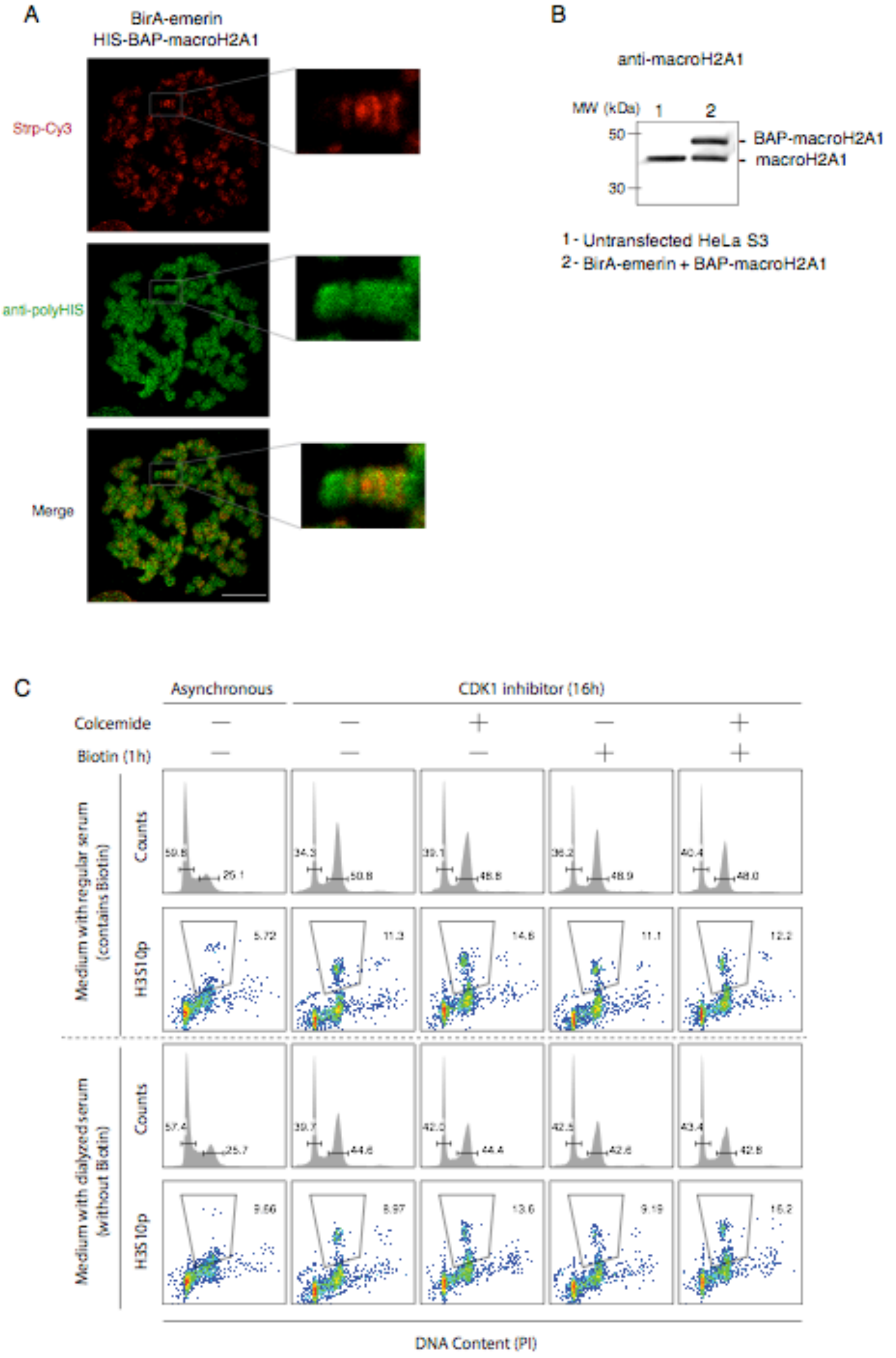

S3: Distribution and protein levels of BAP-macroH2A1 along with FACS profile of HeLa S3 clone co-expressing BirA-emerin and BAP-macroH2A1 with and without biotin.

(A) Homogenous distribution of BAP-macroH2A1 histone on chromosome scale. HeLa S3 clone co-expressing BirA-emerin and BAP-macroH2A1 were biotin labeled, subjected to mitotic spreads preparation followed by streptavidin-Cy3 staining to reveal biotin (red) to reveal biotin and anti-His (green) to detect BAP-macroH2A regardless of its biotinylation state. Shown is an example of a mitotic spread and chromosomes at higher magnification. Scale bar 10 μm. **(B)** Similar levels of BAP-macroH2A1 and endogenous macroH2A1. Western blot analysis performed on nuclei from HeLa S3 cells (1) and HeLa S3 cells co-expressing BirA-emerin and BAP-macroH2A1 (2). **(C)** HeLa cells were grown either in the media containing regular fetal bovine serum (FBS) or in the media containing dialyzed FBS for two weeks. Cells were then synchronized in late G2 phase using CDK1 inhibitor for 16 hours. Cells were pulsed with biotin for 1 hour and then released in to colcemide for another 45 min to block them in metaphase. As control, cells from both regular and dialyzed serum media without biotin pulse were also released into the colcemide. Cells were fixed and stained with propidium iodide (PI) for DNA content and histone H3 serine 10 phospho (H3S10p) antibody as a mitotic marker. The numbers on PI profile represent the number of cells in G1 and G2 phases, respectively, while the gated population in H3S10p stained samples represents the number of H3S10p positive cells.

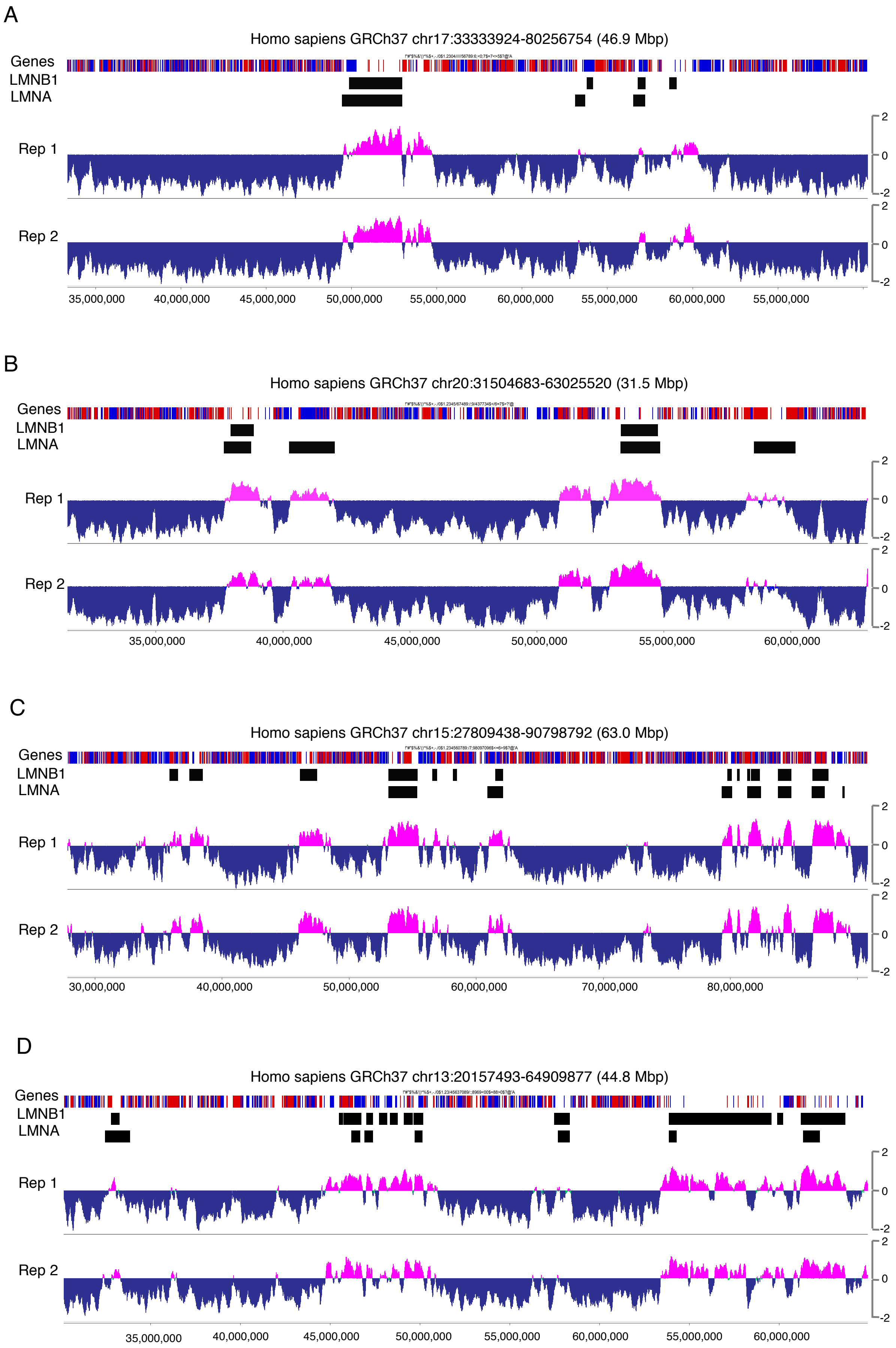
A
Homo sapiens GRCh37 chr17:33333924-80256754 (46.9 Mbp)
Genes
LMNB1
LMNA
Rep 1
Rep 2
2
0
-2
35,000,000
40,000,000
45,000,000
50,000,000
55,000,000
60,000,000
55,000,000
60,000,000
55,000,000
B
Homo sapiens GRCh37 chr20:31504683-63025520 (31.5 Mbp)
Genes
LMNB1
LMNA
Rep 1
Rep 2
35,000,000
40,000,000
45,000,000
50,000,000
55,000,000
60,000,000
C
Homo sapiens GRCh37 chr15:27809438-90798792 (63.0 Mbp)
Genes
LMNB1
LMNA
Rep 1
Rep 2
30,000,000
40,000,000
50,000,000
60,000,000
70,000,000
80,000,000
D
Homo sapiens GRCh37 chr13:20157493-64909877 (44.8 Mbp)
Genes
LMNB1
LMNA
Rep 1
Rep 2
35,000,000
40,000,000
45,000,000
50,000,000
55,000,000
60,000,000
55,000,000
60,000,000

S4: **Exemplary regions demonstrating the enrichment of biotinylated BAP-macroH2A1 across HeLa LADs.**

(A) chr17:33333924-80256754, **(B)** chr20: 31504683-63025520, **(C)** chr15: 27809438-90798792, **(D)** chr13: 20157493-64909877 on human genome (hg19/GRCh 37). The genome was divided into 10kb bins and the PUB-NChIP-seq signal relative to input was calculated. For visualisation smoothing correction of 5 adjacent probes was applied.

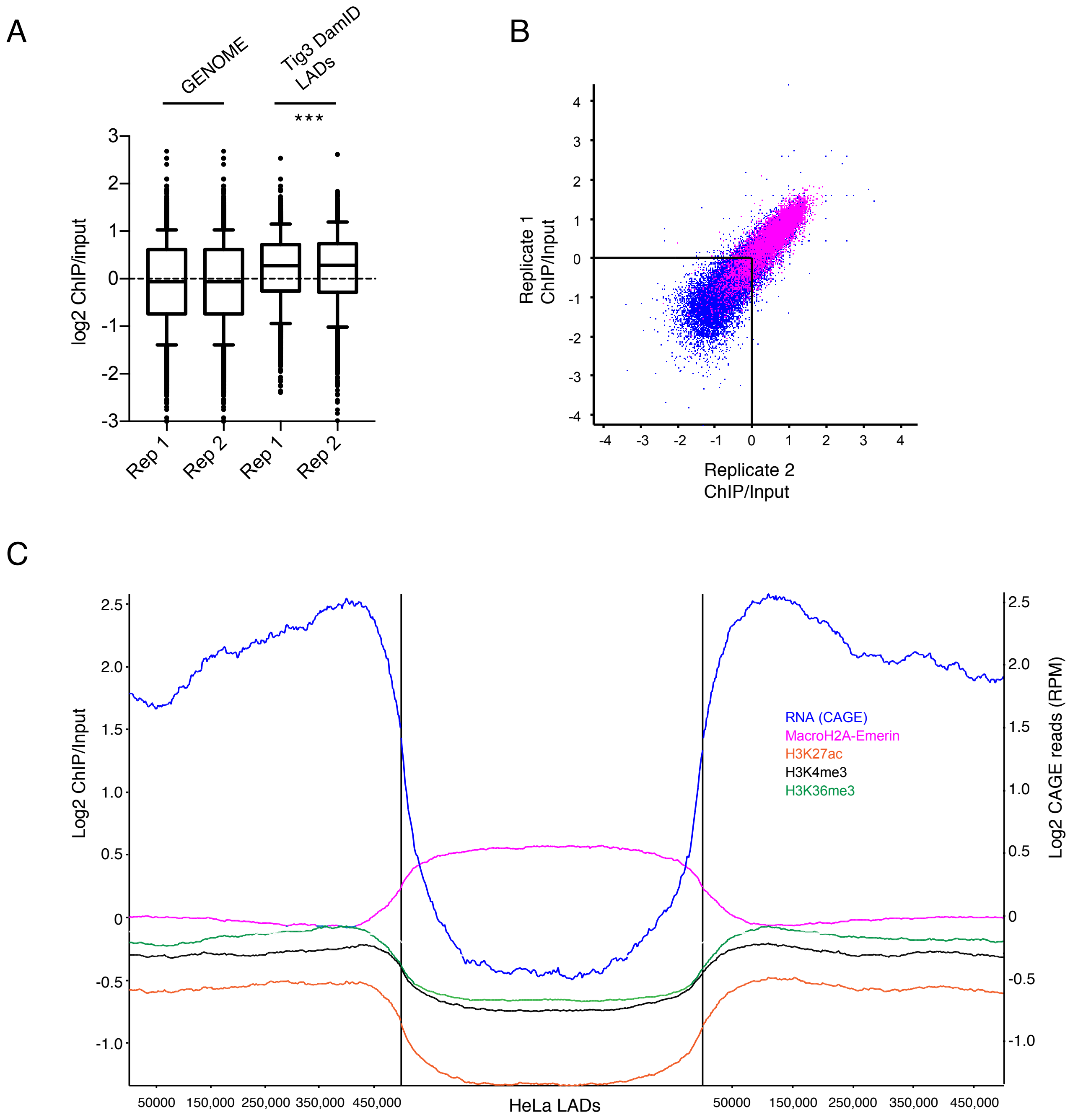

S5: Comparison of PUB-NChIP-seq with DamID and histone marks enriched in transcriptionally active chromatin

(**A**) PUBnChIP signal is enriched at DamID LADs. We obtained Tig3 human fibroblast, DamID defined LADs and measured the enrichment of PUB-NChIP-seq signals in 10kb genomic fragments across the whole genome or at DamID Tig3 LADs. Monte carlo simulations were used to test for statistical significance between the genome wide average and Tig3 (human fibroblast cell line) DamID LADs (p>0.001). (**B**) Overlap of LADs identified by Lamin ChIP-seq and PUB-NChIP-seq. 10kb genome fragments scored for nuclear lamina enrichment signal as measured by PUB-NChIP-seq for genomic regions that do not overlap HeLa LADs (blue)

and those which coincide with HeLa LADs (magenta). The vast majority of LAD overlapping regions show positive PUB-NChIP-seq enrichment demonstrating the strong genome wide correlation between HeLa LADs previously determined by lamin ChIP-seq and PUB-NChIP-seq. **(C)** Enrichment of PUB-NChIP-seq signal and depletion of active histone modifications at LADs generated by DamID in Tig3 cells. CAGE data also shows an inverse relationship with PUB-NChIP-seq signal.

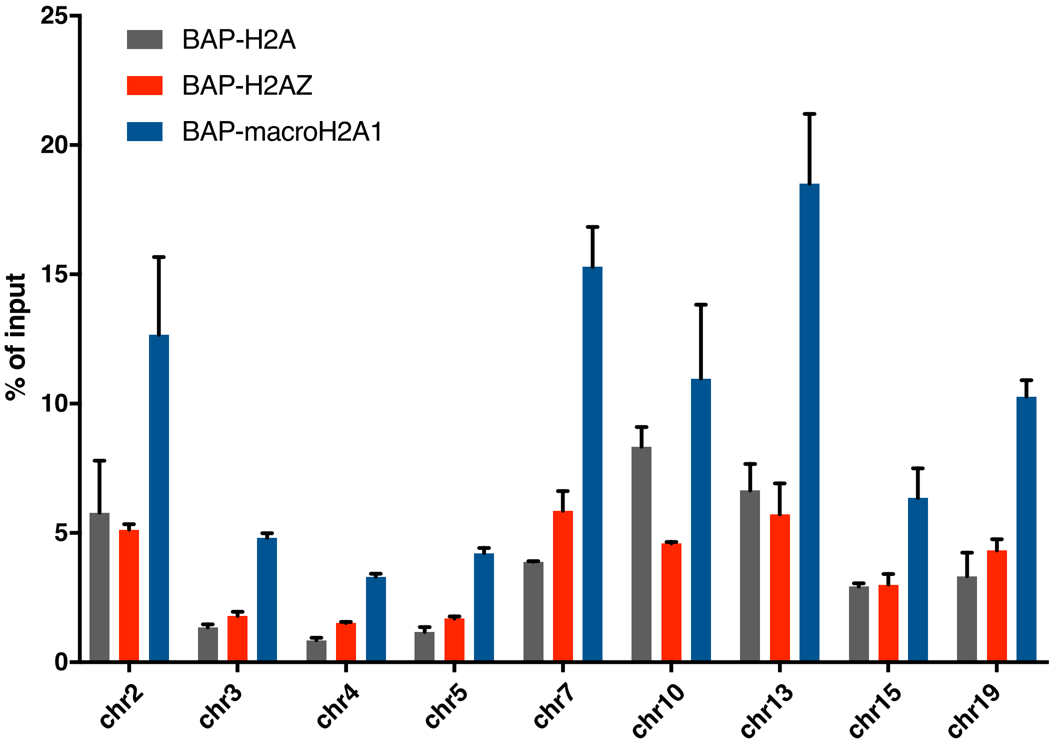

S6: **PUB-NChIP-qPCR analysis of HeLa cells coexpressing BirA-emerin and different variants of histone H2A.** HeLa cells coexpressing BirA-emerin and BAP-H2A or BAP-H2AZ or BAP-macroH2A1 were biotin labelled for 1h and subsequently harvested. PUB-NChIP was performed as described in Materials and Methods and quantitative real time PCR (qPCR) was performed using primers amplifying different regions of chromosomes 2, 3, 4, 5, 7, 10, 13, 15, 19. The bars represent % of input in each sample ± SEM from 2 independent experiments

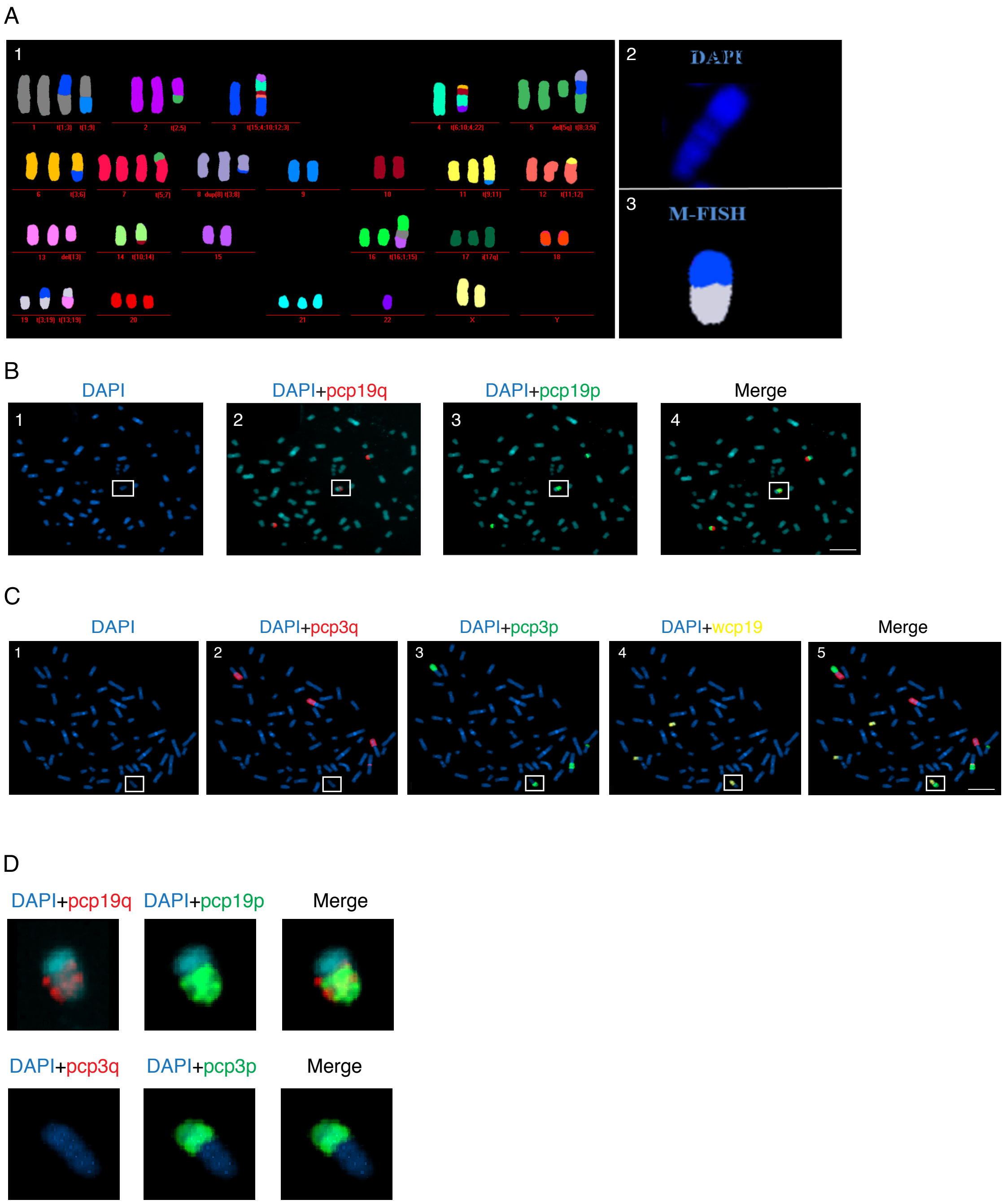

S7: **Karyotypic characterization of the HeLa BirA-emerin and BAP-macroH2A1 expressing clone.**

(**A**) M-FISH karyogram showing hypotriploid karyotype (1). DAPI and M-FISH staining of der(19)t(3;19) chromosome (2 and 3). (**B**) Two color-FISH with partial chromosome painting (pcp) probes for chromosome 19 short (pcp19p; shown green) and long (pcp19q; red) arms. Scale bar 10 µm. (**C**) Three color FISH staining with pcp probes for chromosome arms 3p (pcp3p; green) and 3q (pcp3q; red) and whole chromosome painting (wcp) probe for chromosome 19 (wcp19; yellow). Spreads were counterstained with DAPI (blue). Scale bar 10 µm. (**D**) Details of der(19)t(3;19) chromosome as characterized by two color-FISH and DAPI staining.

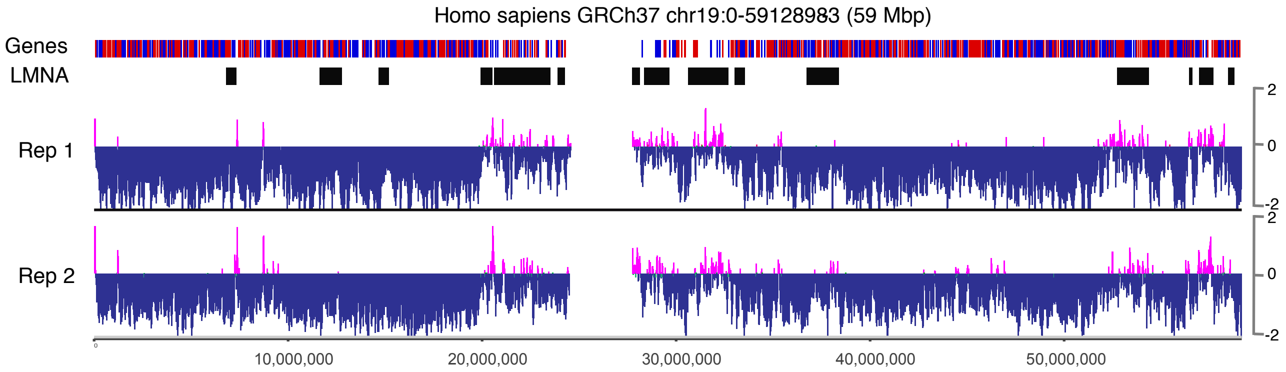

S8: Comparison of chr19 LADs from DamID dataset versus PUB-NChIP-seq.

PUB-NChIP-seq data showing the weak nuclear lamina interaction strength in HeLa cells of chr19, which is similar to that observed by single cell DamID data in KBM7 cells.

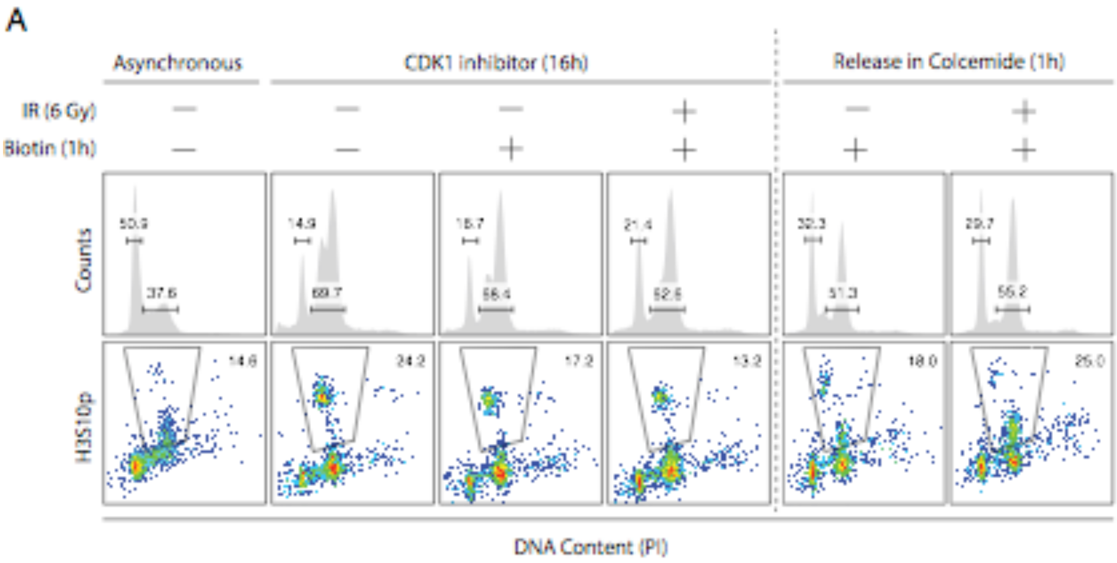

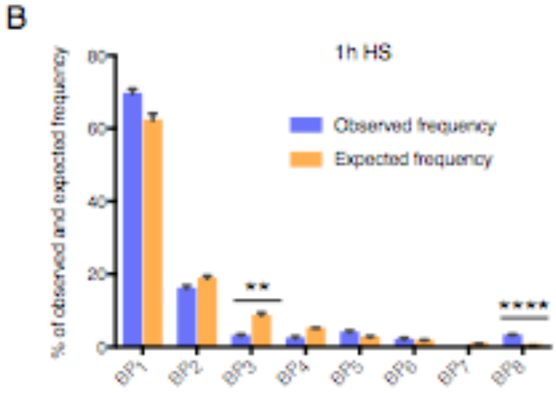

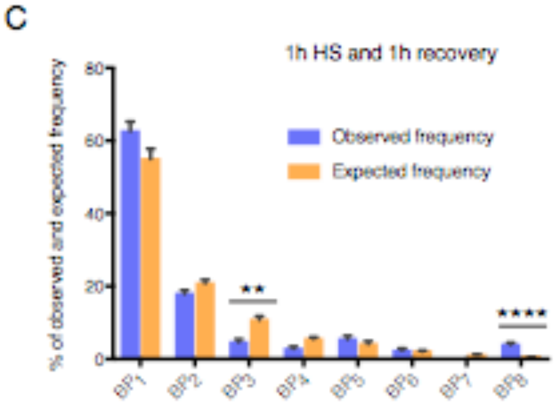

S9: FACS profile of HeLa cells coexpressing BirA-emerin and BAP-macroH2A1 with and without ionizing radiation and analysis of BPs after stress exposure (heat shock).

(A) HeLa cells were synchronized in late G2 phase using CDK1 inhibitor for 16 hours. Cells were treated with ionizing radiation (5Gy), were pulsed with biotin for 1 hour and then released in to colcemide for another 45 min to block them in mitosis. Cells were fixed and stained with propidium iodide (PI) for DNA content and histone H3 serine 10 phospho (H3S10p) antibody as a mitotic marker. The numbers on PI profile represent the number of cells in G1 and G2 phases, respectively, while the gated population in H3S10p stained samples represents the number of H3S10p positive cells. (**B & C**) Observed and expected frequencies of BP for individual cells grown in different experimental conditions are indicated. ** p<0.01; **** p<0.0001, determined by Chi-square test. Data are presented as means ± SEM of three independent experiments.

Supplemental Methods:

Multiplex flourescence in-situ hybridization

M-FISH was performed as described by (Geigl et al., 2006). Briefly, seven pools of flow-sorted human whole chromosome painting probes were amplified and directly labeled using seven different fluorochromes (DEAC, FITC, Cy3, Cy3.5, Cy5, Cy5.5, and Cy7) using degenerative oligonucleotide primed PCR (DOP-PCR). Metaphase chromosomes immobilized on glass slides were denatured in 70% formamide/2xSSC pH 7.0 at 72°C for 2 minutes followed by dehydration in a degraded ethanol series. Hybridization mixture containing combinatorially labeled painting probes, an excess of unlabeled cot1 DNA, 50% formamide, 2xSSC, and 15% dextran sulfate were denatured for 7 minutes at 75°C, pre-annealed at 37°C for 20 minutes and hybridized at 37°C to the denaturated metaphase preparations. After 48 hours, the slides were washed in 2xSSC at room temperature for 3x 5 minutes followed by two washes in 0.2xSSC/0.2% Tween-20 at 56°C for 7 minutes, each. Metaphase spreads were counterstained with 4.6-diamidino-2-phenylindole (DAPI) and covered with antifade solution. Metaphase spreads were recorded using a DM RXA epifluorescence microscope (Leica Microsystems, Bensheim, Germany) equipped with a Sensys CCD camera (Photometrics, Tucson, AZ). Camera and microscope were controlled by the Leica Q-FISH software and images were processed on the basis of the Leica MCK software and presented as multicolor karyograms (Leica Microsystems Imaging solutions, Cambridge, United Kingdom).

Supplementary table 1

List of primers used in Figure 5F

**Name of primer**	**Sequence**
chr3p_1-F	GAAGGGAGAGGAACAGGTGC
chr3p_1-R	CAGGATCCTCCTGCCTCCTA
chr3p_2-F	GAAACCCCTGGTGGTGAAGT
chr3p_2-R	AGCTGCACTCGGTTACTTTGA
chr3p_3-F	AGGGTGCAACCATGCTCATT
chr3p_3-R	AGAACCAATCCAAAAGGCTTGC
chr19p_1-F	TGTTTGATCTCACGCACTCAC
chr19p_1-R	GCGGTTAACGAGTGGGTTAC
chr19p_2-F	TCAGCTGTACCCATGTTGACTA
chr19p_2-R	TAACACACATCCACTATGGGG
chr19p_3-F	GTTTCAGTCCAGATGCGTAGTGAT
chr19p_3-R	TAGGAGCACTTCACTCAGTGGC
chr2-F	GAGGCCTCCTACTCTAGGTCA
chr2-R	GGGCCCTAGTTTGGCATTCA
chr7-F	AATATGGAAGAGAAAGTGGGGGAAA
chr7-R	GATTGCAAGACCCACATTTTTGC

Supplementary table 2

List of primers used in Supplemental Figure S6

**Name of primer**	**Sequence**
chr2-F	GAGGCCTCCTACTCTAGGTCA
chr2-R	GGGCCCTAGTTTGGCATTCA
chr3-F	GAGGCCTCCTACTCTAGGTCA
chr3-R	GGGCCCTAGTTTGGCATTCA
chr4-F	TTCTGGTGACACTCGCTGAC
chr4-R	CCCATATGCCCGAGGGTAAC
chr5-F	ACCAAGAGCGTTTTCAGCCT
chr5-R	GCTACACTGCTCTGGGTGTT
chr7-F	AATATGGAAGAGAAAGTGGGGGAAA
chr7-R	GATTGCAAGACCCACATTTTTGC
chr10-F	TGCCTCCTGGAAACTGTGAC
chr10-R	AGCAAAGGCTGCTGGATGAT
chr13-F	GGCCACGATTGAGTGAATGC
chr13-R	TGCTGCCAAAGAGAAGCTCA
chr19-F	TCAGCTGTACCCATGTTGACTA
chr19-R	TAACACACATCCACTATGGGG
